# NFFinder: an online bioinformatics tool for searching similar transcriptomics experiments in the context of drug repositioning

**DOI:** 10.1093/nar/gkv445

**Published:** 2015-05-04

**Authors:** Javier Setoain, Mònica Franch, Marta Martínez, Daniel Tabas-Madrid, Carlos O. S. Sorzano, Annette Bakker, Eduardo Gonzalez-Couto, Juan Elvira, Alberto Pascual-Montano

**Affiliations:** 1 National Center for Biotechnology-CSIC, Madrid, Spain; 2 The Children Tumor Foundation, New York, USA; 3 Perkin Elmer España, S.L., Madrid, Spain

## Abstract

Drug repositioning, using known drugs for treating conditions different from those the drug was originally designed to treat, is an important drug discovery tool that allows for a faster and cheaper development process by using drugs that are already approved or in an advanced trial stage for another purpose. This is especially relevant for orphan diseases because they affect too few people to make drug research *de novo* economically viable. In this paper we present NFFinder, a bioinformatics tool for identifying potential useful drugs in the context of orphan diseases. NFFinder uses transcriptomic data to find relationships between drugs, diseases and a phenotype of interest, as well as identifying experts having published on that domain. The application shows in a dashboard a series of graphics and tables designed to help researchers formulate repositioning hypotheses and identify potential biological relationships between drugs and diseases. NFFinder is freely available at http://nffinder.cnb.csic.es.

## INTRODUCTION

Drug repositioning or repurposing consists in identifying and using known drugs that can target diseases other than those for which they were originally designed (1). Working with drugs having known properties can significantly reduce the cost and the time needed to enter into clinical phases. In the past, most of repositioning has been done by accident. However, new technologies are allowing the evaluation of more and more existing drugs to check whether they are suitable to treat different disorders. Two main strategies, drug and disease based, have been adopted to find new drug–disease interactions (2). Drug-based strategies look for chemical properties, molecular activity or structure similar to other known therapeutic drugs already used to treat a particular disease in which we are interested. Disease-based strategies look for diseases showing pathological mechanisms similar to the disease relevant for us. This second strategy assumes that therapeutic drugs useful treating similar diseases might also be effective against our target disease. The most effective strategies might involve drug- and disease-based approaches (1). Another strategy addresses drug repositioning when researching with genetic disorders showing alterations in gene expression patterns that may be reverted by drugs. This last approach opens up the doors to mine the existing gene expression studies related to diseases and drugs, finding relationships between them as a starting point to drug repurposing. The widespread use of techniques like DNA microarrays and Next-Generation Sequencing to measure gene expression have allowed the creation of databases containing information about those expression profiles in different studies with the purpose of centralizing all such information to show it in a more homogeneous way. The most prominent examples of this type of repositories are NCBI's Gene Expression Omnibus (GEO) (3) and EMBL-EBI's ArrayExpress (4), both containing >50 000 functional genomics experiments. Other databases like the Broad Institute's Connectivity Map (CMap) (5) and DrugMatrix (6) collect highly specific expression data from samples treated with drugs and other chemicals, providing some more direct correlations between the expression profiles and the compounds.

There exist methods that use transcriptional data to infer connections between drugs and diseases. CMap, in addition to hosting a collection of expression data, allows to query signatures against all its data and, with simple pattern-matching algorithms very similar to those used in Gene Set Enrichment Analysis (7), it enables the discovery of functional connections between drugs, genes and diseases. Cha *et al*. (8) used part of the data contained in CMap to extract significant differentially co-expressed gene modules, associating them to drug response profiles and constructing a drug–drug network allowing finding drugs having the same target proteins and novel drug relations. In the Combinatorial Drug Assembler (CDA) (9), the authors created a system in which up- and down-regulated genes from a query are processed to perform a signaling pathway gene set enrichment analysis. Then, a signaling pathway and drug set enrichment analysis is performed against the CMap database, and similarities between these analyses and the one performed to the query are searched. The CDA program generates lists of drugs showing similar expression patterns. Finally, other more general tools to perform comparisons between gene expression profiles have also been developed (10,11).

We present NFFinder, a bioinformatics web-based tool for creating hypotheses in drug repositioning initially developed in the context of orphan diseases like Neurofibromatosis (NF) and later generalized to any other disorder. These diseases do not usually draw the attention of the pharmaceutical industry because they affect only a small percentage of the population and this provides little financial incentive to develop and commercialize new specific drugs. A cheaper and faster alternative to the traditional drug discovery pipeline, in which a drug is expected to cost many hundreds of millions over 10–15 years, is needed in order to boost the discovery of therapeutics for those rare diseases.

NFFinder tool requires as input a differential analysis experiment consisting of two lists of up- and down-regulated genes or a list of microRNAs present in the experiment. A selective query against our database, which contains curated DataSets from GEO, CMap and DrugMatrix, is performed to compare the input with the selected signatures from our database using a method based on the one used in MARQ (12). The tool returns experiments with similar or opposite gene profiles, in association with a correlation score and a statistical significance value. The experiments contained in our database have been processed with data mining tools like MetaMap (13) to enrich them with terms related to drugs, diseases and expert scientists. We have also taken advantage of the information about drugs and chemical compounds present in CMap and DrugMatrix. Lastly, the results of the comparison are visually presented in a highly graphical environment allowing the user to explore, select, filter and display result views centered in drugs, diseases, gene expression signatures and expert authors. Depending on the input genes and the options selected, users will be able to explore a huge database of experiments with phenotype similar or opposite to the input one and to discover multiple drugs with similar effect or with an opposed profile to the one's particular disease.

## MATERIALS AND METHODS

**Figure 1.**
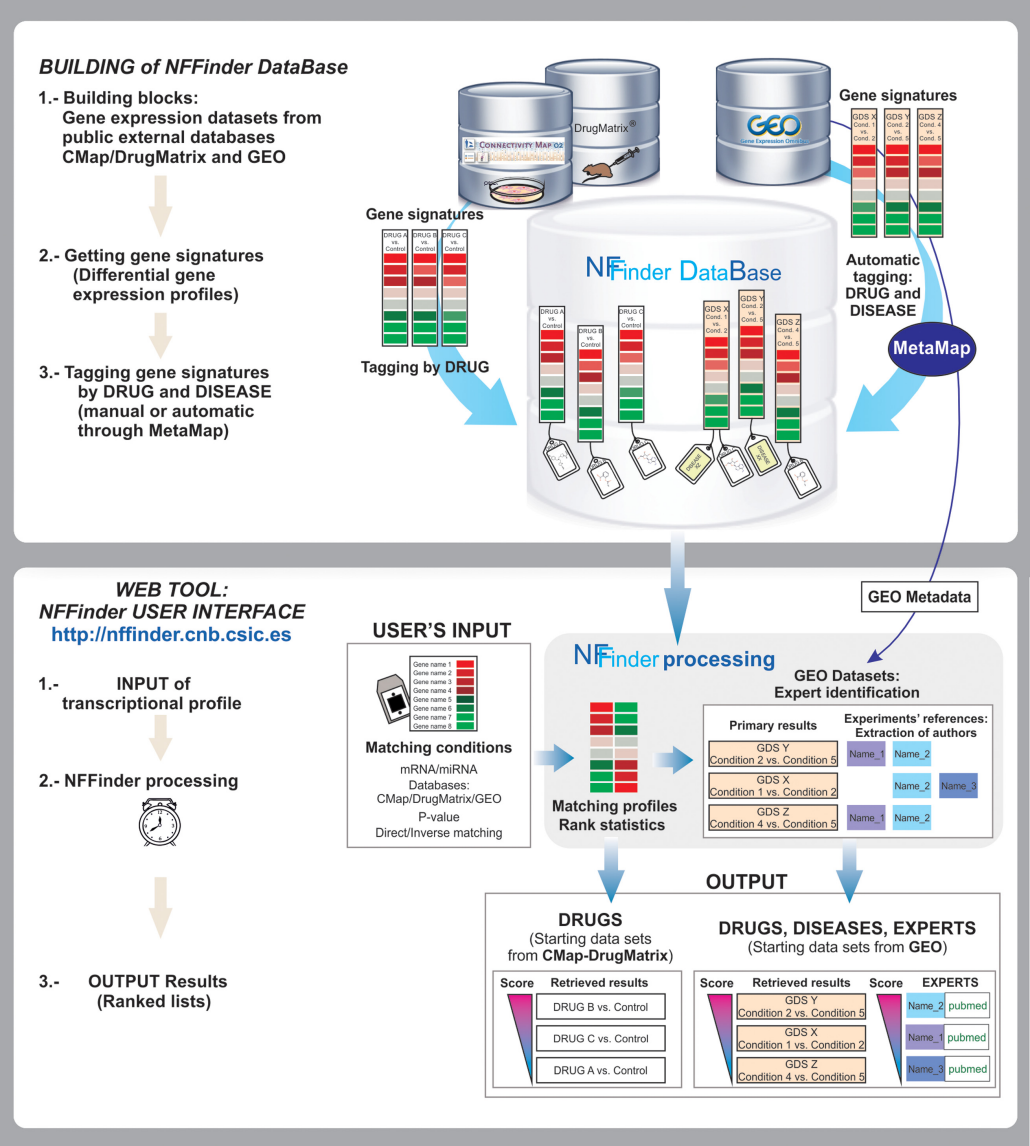
General schema of NFFinder functionality.

As a matching tool of gene expression profiles, NFFinder is based on a huge internal database containing thousands of gene signatures tagged with drug- and disease-related terms (Figure 1, upper panel). NFFinder compares a user's input phenotype of interest, in the form of two lists of up-regulated and down-regulated genes (as Gene Symbols) or a list of miRNAs present in the experiment, with the list of NFFinder database gene signatures (Figure 1, lower panel). As a very computationally demanding task, the profile comparison processing could take up a few hours to finish depending on the selected matching conditions. Once NFFinder gets the list of gene signatures similar—directly or inversely—to the input phenotype, it analyzes the sources of those signatures to extract a list of experts related to that phenotype. The retrieved information is presented to the users as a series of graphics and tables facilitating the exploration of relationships among drugs, diseases and the input phenotype in search of potential new uses for known drugs. Figure 1 shows a general overview of how NFFinder works and the details are exposed in the following subsections.

### Signature database construction

In order to build the gene signature databases we mined three different public databases with transcriptomic data in them: GEO, CMap and DrugMatrix. We obtained 16 432 gene expression signatures by processing 3254 GEO DataSets. We used R in order to automatically generate the gene signatures from the GEO DataSets. With GEOQuery (14) we loaded each individual DataSet and used Limma (15) to perform the differential expression analysis between every pair of classes inside each experimental condition. We then sorted the genes by their *t-value* (or fold-change if there are not enough sample replicates to perform the statistical analysis) in order to obtain a gene signature. The direction of the comparison was arbitrarily set unless one of the classes was identifiable as a control or the condition was identifiable as a time series. The automatic analysis of differential gene expression could introduce spurious comparisons that the researcher should discard after inspecting the results. In addition to the GEO DataSets’ signatures, we added 6100 signatures from CMap and 5288 more from DrugMatrix for a total of 27 820 different gene signatures.

### Drug-related and disease-related terms tagging

Tagging CMap and DrugMatrix signatures with a drug-related term was a straightforward process given that these databases contain simple experiments of specific compounds on different cell lines or rat tissues, respectively. Gene signatures extracted from GEO were more difficult to catalog automatically since every DataSet contains different experimental conditions and classes. We solved that problem by using a third party natural language analysis tool specialized in biomedical texts: MetaMap (13). MetaMap uses a knowledge intensive approach based on symbolic, natural language processing and computational linguistic techniques.

Feeding the GEO DataSet descriptions to MetaMap we obtained lists of terms grouped by class. In a first step, we identified the classes that contained terms related to drugs or terms related to diseases and conditions. All the terms found in all GEO DataSet descriptions were filtered discarding those that did not make sense or were not helpful in our context. The list of meaningless terms was manually created after examination of our own results during the tool's testing process. Finally, the so-constructed list of terms was used to tag every gene signature obtained from each GEO DataSet with a set of one or more drug-related terms and/or a set of one or more disease-related terms extracted from the semantic analysis of its description. This allowed us to establish relationships not only between a gene signature and a group of diseases/drugs, but also among drugs, diseases and between drugs and diseases. Remark that NFFinder database does not include a comprehensive list of drug–disease relationships. Only the drug or disease terms tagging gene signatures from comparisons of samples enclosed in retrieved GEO DataSets will appear in the lists of drugs or diseases.

### Signature comparison

In order to find signatures in NFFinder's database that are similar or opposed to the input, we used a methodology similar to the one employed by MARQ (12), in which rank statistics is used to assign a score and a *P*-value to two lists of genes: up-regulated and down-regulated. The score is computed using a weighted Kolmogorov–Smirnov-like statistic, obtaining a similarity value normalized from 0 to 100. The significance of such score is determined using a random permutation test.

### miRNA to mRNA translation

As we mentioned earlier, NFFinder takes mRNA or miRNA data as input but the signature comparison is performed at mRNA level. In order to use miRNA as input, we need to transform a list of miRNAs up-regulated in a specific condition into a NFFinder standard mRNA input. With that purpose, we used four different databases of experimentally verified interactions between miRNAs and mRNAs (16–19) and a dictionary to translate every miRNA nomenclatures into miRBase format (20). Since almost every miRNA–mRNA interaction found to date is an inhibiting one, from a list of up-regulated miRNAs we could infer a list of down-regulated genes. Likewise, from a list of down-regulated miRNAs we could infer a list of up-regulated genes. With those lists we proceed as described earlier in this section.

### Expert identification

With the aim of promoting collaborations and further research, NFFinder output highlights the authors with expert knowledge in biological processes potentially related to our experiment. In order to associate phenotypes with experts we devised a strategy based on references. We begin with the list of gene signatures returned by NFFinder related to an input phenotype of interest. We take those obtained from GEO DataSets and extract the authors of the papers in the ‘citation’ section of the source DataSet. For every gene signature associated with a DataSet that cites a paper by a particular author we increase her or his score by one. In this way, when NFFinder assigns a score of *N* to an author it means that the author has been involved in the experiments of *N* gene signatures similar to the input phenotype.

### Improvements of NFFinder over similar tools

A common list of differentially expressed genes of gastric cancer ((21) Supplementary Table S2) was submitted to web-based tools CMap, CDA and NFFinder (inversely profiled against CMap/DrugMatrix databases) in order to compare the behavior of these three tools. Supplementary Table S1 summarizes the results according to CMap top ranking. The three methods identify four compounds (LY-294002, Trichostatin A, Tanespimycin and Vorinostat) with significant *P*-value lower than 0.005. Due to the multi-signaling pathway association with compounds accomplished by CDA, this tool did not find Resveratrol and Trifluoperazine as significant compounds. However, CMap and NFFinder, without that constraint, also identified Resveratrol and Trifluoperazine. Claerhout *et al*. (21) determined through gene expression analysis and experimentally validated the histone deacetylase inhibitor Vorinostat as a new therapeutic drug for gastric cancer treatment. Although that study proves that CMap, CDA and NFFinder are suitable tools setting up working hypotheses, NFFinder shows clear improvements over the other two methods. Concerning the number of profiles to compare, the huge NFFinder database includes not only the CMap/CDA profiles but also those derived from DrugMatrix and curated GEO DataSets. This upgrade of NFFinder also allows searching not only for drugs but also for diseases and experts. Dealing with web functionality, NFFinder is easier to set up and use than CMap/CDA. NFFinder does not require any registration to submit queries, whereas CMap does and CDA requires an e-mail address. Unlike CMap, which constrains queries to specific probes, NFFinder allows to query the database with gene symbols. NFFinder gives a clear rank of results including details from profiles of comparison, whereas CDA web interface shows only part of results with little additional information. NFFinder web interface is richer allowing users to examine results with different interactive tools and displays.

## NFFinder USE CASE AND OUTPUT DESCRIPTION: LOOKING FOR DRUGS TO KILL MPNST CELLS

NF is an autosomal dominant disease caused in humans by deficiencies in one of the neurofibromin genes, NF1 or NF2. Patients may develop different abnormalities in skin, eyes, skeleton, cardiovascular, endocrine and nervous systems. In the peripheral nervous system, disorders typically manifest as benign neurofibromas that eventually may degenerate to malignant peripheral nerve sheath tumors (MPNST). NF has been classified into three distinct types: NF1, NF2 and Schwannomatosis. The most common one, NF1, occurs in 1:3000 births (22,23) and is considered a rare disease.

Searching for drugs to revert the phenotype of NF patients to a healthier one constitutes one of the most important applications of NFFinder. To illustrate this functionality of the tool we looked for antitumor drugs to kill the MPNST cell line ST88–14 derived from a neurofibromin-deficient patient.

### Case 1 INPUT against drug databases

Up- and down-regulated genes were obtained from microarray analyses comparing MPNST cells versus Normal Human Schwann Cells (24) (Supplementary Figure S1a Case 1). The list of differentially expressed genes was inversely profiled against CMap and DrugMatrix databases.

### Case 1 OUTPUT

The search with NFFinder retrieved 775 entries containing 391 different compounds. The selection of 30 drugs with higher score plus the 10 more abundant (four entries or more in the whole list) resulted in 32 compounds involved in treating cancer (56%), NF (12%), neurological disorders (12%), skin-related diseases (12%) and other benign neoplasia (3%) (Supplementary Figure S1b Case 1). Forty percent of the total amount of drugs to treat cancer serve to treat glioblastoma and other nervous system malignancies. Being effective against glioblastoma (25), Trichostatin A (TSA) is the best result appearing 133 times in total (17%) and 23 times among the 30 drugs with higher score (77%). The histone deacetylase inhibitor TSA, with a wide range of epigenetic activities, is considered a multifunctional anticancer drug. Besides glioblastoma, TSA shows effectiveness against other malignancies such as breast cancer or esophageal squamous cell carcinoma by blocking cell proliferation and triggering apoptosis (26).

In a recent work with a NF1/SUZ12 null cell line, De Raedt *et al*. (27) took advantage of epigenetics to kill MPNST cells and to shrink tumors through the combination of two synergy-acting compounds, PD-0325901 (PD-901) and JQ1. PD-901 acts as RAS-MEK pathway inhibitor counteracting the neurofibromin deficiency. JQ1 compensates deficiencies in SUZ12 or DEE, components of the polycomb repressive complex (PCR2). The absence of SUZ12 is associated with histone modifications that activate gene expression recruiting bromodomain proteins and other transcription factors. JQ1, as a bromodomain inhibitor, promotes the condensation of chromatine and gene silencing. Mutations or deletions of *SUZ12* (or *DEE*) are usually linked to ablations in *NF1* gene. The double deficiency in NF1 and SUZ12 cooperates to develop MPNSTs and other types of tumors. In the cell line ST88–14, also depleted in SUZ12 (logFC = −4.20; adjPVal = 2.16E-20), TSA could be involved, besides in the epigenetic regulation, in the blocking of the RAS pathway as PD-901/JQ1 does in the 90–8TL cell line. In fact, TSA inhibits the RAS-regulated pathways PI3K/Akt and ERK1/2 (26).

To test whether TSA replaces the combination of PD-901 and JQ1 to kill MPNST cells, we carried out a second analysis illustrating the application of NFFinder to look for drugs with similar effect to other compounds already tested.

### Case 2 INPUT against drug databases

Differentially expressed genes were obtained comparing control DMSO treated cells versus PD-901/JQ1 treated cells (Supplementary Figure S1a Case 2). This independent gene input was directly profiled against CMap and DrugMatrix.

### Case 2 OUTPUT

NFFinder also retrieved TSA as best result with 67 entries in total (14%) and 19 entries among the 30 with higher score (63%). Interestingly, we have found TSA as common best result using as input independent gene profiles including 20 (Case 1) and 23 (Case 2) genes from RAS pathway signature (27) (Supplementary Figure S1). This result reinforces that TSA, with a double effect targeting epigenetic modifications and RAS pathway, could be an interesting drug to test its effectiveness to treat MPNST derived from NF1 deficiency. Moreover, this result shows that NFFinder is a robust and powerful tool to pose working hypotheses in the context of therapeutics of NF and other rare diseases. Details of Case 2 analysis and the comparison with Case 1 are shown in Supplementary Figure S1.

Inspection of GEO database constitutes a complementary approach to look for drugs. Retrieved GEO experiments involving drugs or diseases with already known therapeutics drive to new hypotheses for drug repurposing.

### Cases 1 and 2 INPUT against GEO database

To illustrate the complementary application of NFFinder looking for drugs, we tested both case gene inputs against GEO database and details of the retrieved experiments are shown in Supplementary Figure S1.

### Cases 1 and 2 OUTPUT

The number of comparisons that make sense (appropriate) among samples in GEO accessions varies between 70 and 80% in the 200 results with higher score. Not surprisingly, most part of comparisons referred to cancer disease (at least 55% in the 200 results with higher score) and two comparisons from GDS2736 accession DataSet involved MPNST cells. A careful examination of individual experiments including drugs could suggest any of these compounds as hypotheses for therapeutics of MPNST.

**Figure 2.**
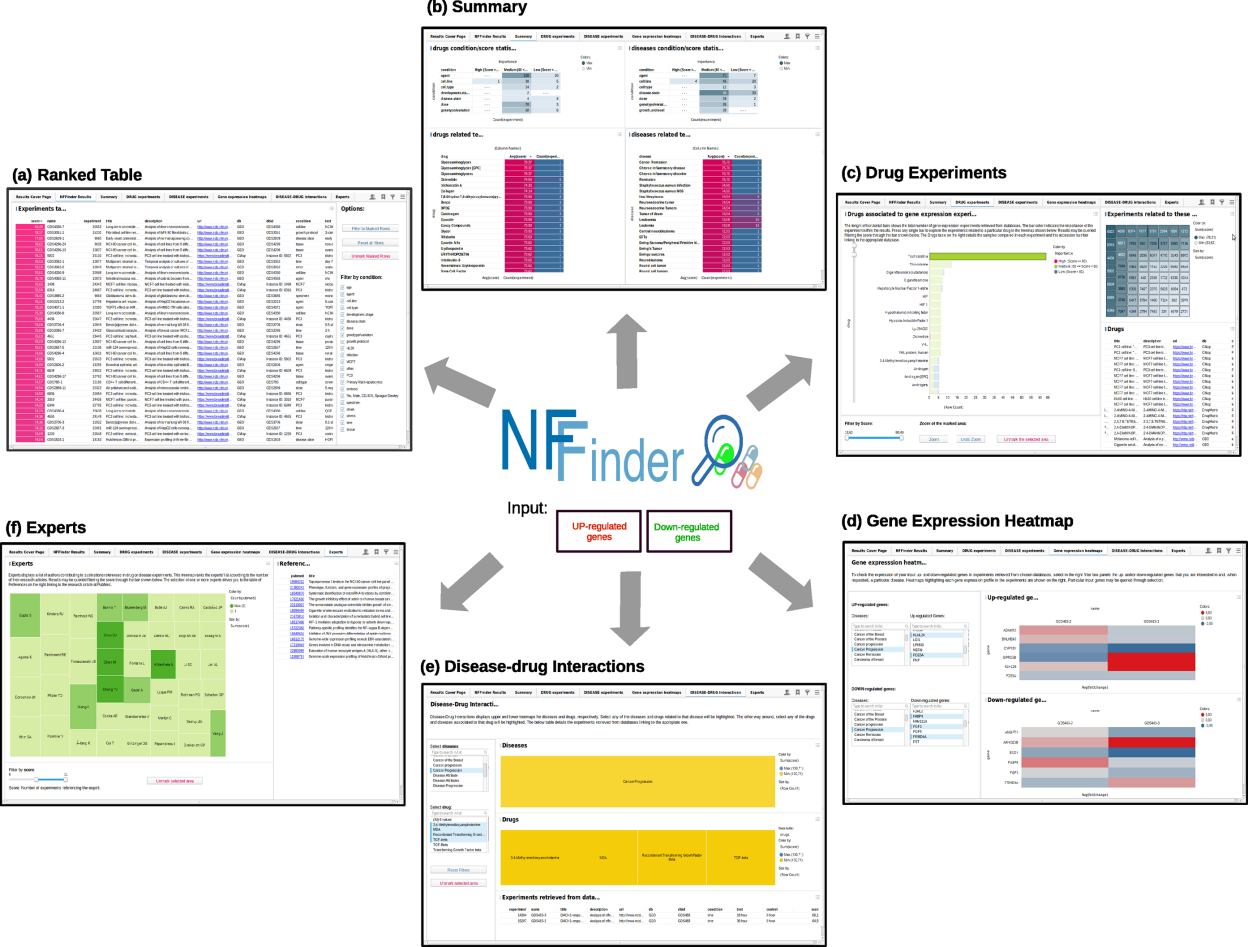
Collage of the output results generated by NFFinder. Case 2 gene profile was used as input against databases GEO and CMap-DrugMatrix by inverse and direct profile matching, respectively. NFFinder provides the following output displays. **(a)** Ranked table of retrieved results. **(b)** Aggregated summary of results. **(c)** Drugs or diseases related to the experiment results. **(d)** Heatmaps to visualize the expression of the input genes in the context of some diseases relevant for users. **(e)** Interactions between drugs and diseases. **(f)** List of authors that contributed to the results of a specific search (experts).

We have included a tutorial in the website with the different possible types of applications of the tool that users can launch and the steps to interpret the results. NFFinder ranked results from GEO and CMap-DrugMatrix are displayed through a friendly interface allowing users to visualize the output from different perspectives such as drugs, diseases, drug–disease interactions and scientific experts (Figure 2).

## TECHNICAL DETAILS

NFFinder was implemented in Python using Django and Django REST framework for the web application back-end and the REST web service respectively. NFFinder's back-end relies on a PostgreSQL database holding experimental data as well as job information. The web interface uses Javascript to create the users’ jobs and track their progress through the REST API. These jobs, written in Python, are executed in a dedicated computing cluster that contains six computing nodes with two Quad-Core Intel Xeon processors each. Front-end and visualizations of results can be pulled out once the job is completed to be visualized with any visualization system. For this project we have used the dashboards and enhanced data visualization capabilities of the TIBCO Spotfire® Analytics platform which have been made accessible by PerkinElmer.

## DISCUSSION

NFFinder is the first web-based application of gene expression profiles comparison oriented to drug repositioning that integrates expression data from GEO, CMap and DrugMatrix, including terms mined from the related metadata using MetaMap that can be related through the experiments. NFFinder application provides a complete environment to match similar gene expression signatures, to integrate different data sources, and to find potential collaborators, and thus constitutes a bona fide data scientist enabling platform. There are some limitations attached to NFFinder and other gene expression signature comparison methods. Remark that reverting a particular gene expression signature related to a disease with a drug having an opposite signature is a naïve approach to drug discovery and repositioning, limited only to a subset of the known diseases, and has to be complemented by experimental studies to confirm the hypotheses generated with our tool. In addition, the set of drugs and diseases included in the databases is limited. Also, results coming from cell lines could not be always extrapolated to *in vivo* tissues. Resulting expression profiles, diseases, drugs, drug–disease interactions and experts from the field are downloadable as tables. The REST web services that run the analyses are completely detached from the interface and can also be used to integrate our tool in other pipelines and visualization platforms.

## NFFINDER AVAILABILITY

This application can be freely accessed at http://nffinder.cnb.csic.es.

## SUPPLEMENTARY DATA

Supplementary Data are available at NAR Online.

SUPPLEMENTARY DATA
