# Supplementary material for: NFFinder: an online bioinformatics tool for searching similar transcriptomics experiments in the context of drug repositioning
Source: Nucleic Acids Res. 2015 May 4;43(Web Server issue):W193–9. doi: 10.1093/nar/gkv445 (PMC4489258; doi:10.1093/nar/gkv445)
Supplement: SUPPLEMENTARY DATA [file supp_43_W1_W193__index.html]

NFFinder: an online bioinformatics tool for searching similar transcriptomics experiments in the context of drug repositioning — NFFinder: an online bioinformatics tool for searching similar transcriptomics experiments in the context of drug repositioning — SUPPLEMENTARY DATA 

# NFFinder: an online bioinformatics tool for searching similar transcriptomics experiments in the context of drug repositioning

## SUPPLEMENTARY DATA

**Files in this Data Supplement:**

- SUPPLEMENTARY DATA
