# Supplementary material for: NFFinder: an online bioinformatics tool for searching similar transcriptomics experiments in the context of drug repositioning
Source: Nucleic Acids Res. 2015 May 4;43(Web Server issue):W193–9. doi: 10.1093/nar/gkv445 (PMC4489258; doi:10.1093/nar/gkv445)
Supplement: SUPPLEMENTARY DATA [file supp_gkv445_nar-00450-web-b-2015-File004.pdf]

Supplementary Table S1. Comparison of results retrieved with CMap (1), CDA (2) and NFFinder.

CMap Name	CMap				CDA[a]			NFFinder		
	Rank[b]	Mean Correlation[c]	n[d]	p-value	Mean Correlation[e]	n[d]	p-value[f]	Average Score[g]	n[h]	p-value[i]
LY-294002	1	-0,53	61	0,000	-0,29	61	0,000	53,89	8	0,001
TRICHOSTATIN A	2	-0,53	182	0,000	-0,53	182	0,000	56,65	125	0,001
RESVERATROL	3	-0,76	9	0,00016	-0,29	9	0,4018	55,11	8	0,001
TRIFLUOPERAZINE	4	-0,61	16	0,00058	-0,18	16	0,2475	54,12	3	0,001
TANESPIMYCIN	5	-0,44	62	0,0008	-0,34	62	0,000	53,69	2	0,001
VORINOSTAT	6	-0,63	12	0,00098	-0,57	12	0,000	56,30	10	0,001

[a]CDA associates multi-signaling pathways with drugs. In order to have comparable data, only results involving the pathway showing the lower p-value for a given drug were taken into consideration.
[b]Ranking of negative scoring drugs included in CMap exportable list of permuted results. The lower p-value fits the top ranking.
[c]Mean of connectivity scores (values between -1 and +1 representing the strength of similarity of drug and user's input gene expression profiles).
[d]Times appearing an individual drug among 6100 instances.
[e]Connectivity Score Mean for a particular negative scoring drug given the multi-signaling pathway showing the lower p-value.
[f]Lower p-value of a singular negative scoring drug.
[g]Average Score gives the mean of score values of the instances of an individual drug. The Average Scores can be examined in the Summary table of NFFinder results. Scores represent the strength of similarity of drug and user's input gene expression profiles in a scale from 0,00 to 100,00.
[h] Times appearing a particular drug among 1635 instances. The number of instances is determined by the user's constraint of p-value lower than 0.005.
[i] Minimum p-value of the instances of a singular drug. Maximum p-value is constrained by the user to 0.005.

1. Lamb,J., Crawford,E.D., Peck,D., Modell,J.W., Blat,I.C., Wrobel,M.J., Lerner,J., Brunet,J.-P., Subramanian,A., Ross,K.N., *et al.* (2006) The Connectivity Map: using gene-expression signatures to connect small molecules, genes, and disease. *Science*, **313**, 1929–35.

2. Lee,J.-H., Kim,D.G., Bae,T.J., Rho,K., Kim,J.-T., Lee,J.-J., Jang,Y., Kim,B.C., Park,K.M. and Kim,S. (2012) CDA: combinatorial drug discovery using transcriptional response modules. *PLoS One*, **7**, e42573.

a

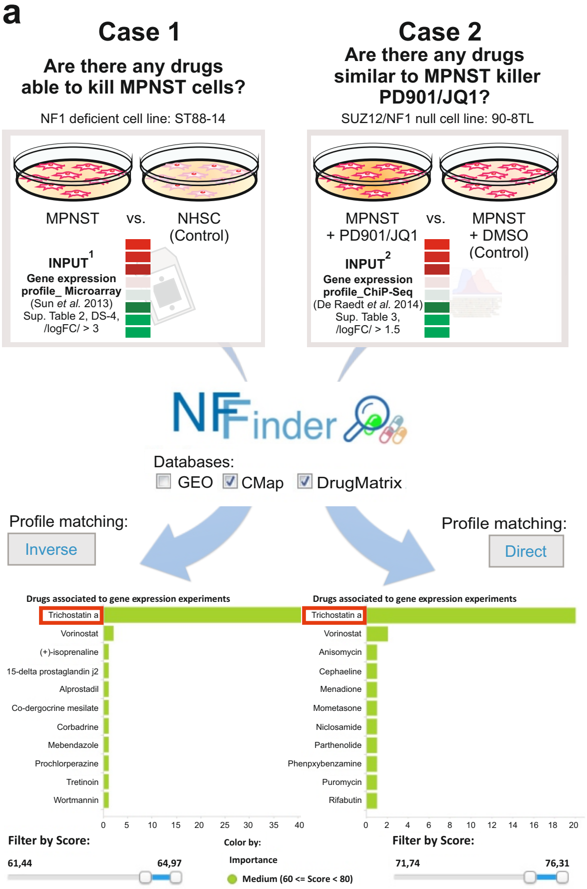

b

	Case 1		Case 2
**OUTPUT CMap-DrugMatrix**	**Inverse profiling**[3] 775 drug experiment entries/ 391different compounds		**Direct profiling**[4] 465 drug experiment entries/ 271different compounds
	**Selection of drugs to inspect: 30 (higher score) + 10 (appear 4 or more times)**		
	32 different compounds	Common drugs: LY-294002, Prochlorperazine, Sirolimus, Trichostatin A, Vorinostat	21 different compounds
	**% of selected compounds related to diseases**[5]		
Cancer	56		62
Neurofibromatosis	12		5
Skin-related	12		14
Neurological disorders	12		24
Other benign neoplasia	3		10
**OUTPUT GEO**	**Direct profiling**[6] 498 comparison entries/ 283 different GDS accessions		**Inverse profiling**[7] 612 comparison entries/ 321different GDS accessions
	**Selection of comparison entries to inspect: 200 (higher score)**		
% of appropiate comparisons	72		81
	**% of selected entries related to diseases/biological processes**		
Cancer	55		58
Skin and epithelial tissue-related diseases	14		17
Hormone effects and differentiation	14		9
Progeria/Senescence	3		3
	**Comparisons involving MPNST samples from GDS2736**		
	MPNST vs. fibroma (Score 69.89) MPNST vs. lipoma (Score 68.76)		MPNST vs. lipoma (Score 53.05)

Supplementary Figure S1. Description of two NFFinder use cases. **a.** Drawing of gene INPUT and main OUTPUT results retrieved from CMap and DrugMatrix databases. **1.** RAS pathway genes: ADAMTS6, ESM1, FOXG1, HAS2, HTR7, IGFBP3, IL8, KIT, LPL, MMP1, PLAU, PTGS2, SEMA3A, SOX9, TLR4 (UP-regulated) and EMP1, NAV3, NRCAM, SCG5, SPP1 (DOWN-regulated). **2.** RAS pathway genes: C11orf41, CYB5R2, DUSP6, EMP1, ESM1, ETV1, ETV5, HAS2, IL1B, ITGA2, LPXN, MMP1, NAV3, NTM, PDCD1LG2, PLAU, PRDM1, PRRX1, SEMA3A, SPRY2, STC1, TFPI, TLR4 (DOWN-regulated). **b.** Summary table of OUTPUT results retrieved from CMap-DrugMatrix and GEO databases. **3.** http://bit.ly/CASE1_CMap-DrugMatrix **4.** http://bit.ly/CASE2_CMap-DrugMatrix **5.** Detailed information in Supplementary Table S2. **6.** http://bit.ly/CASE1_GEO. **7.** http://bit.ly/CASE2_GEO.

Supplementary Table S2. Selected drugs and some diseases treated with them[a].

SELECTED DRUGS[b]	n^c	CANCER	NF	SKIN-RELATED DISEASES	NEUROLOGICAL DISORDERS	BENIGN NEOPLASIA
**CASE 1**						
(+)-ISOPRENALINE	1					
2-AMINO-4-NITROPHENOL	6					
ALLOPURINOL	4				(1)	
ALLYL ALCOHOL	5					
ALPROSTADIL	1					
BENZETHONIUM CHLORIDE	4	(2)				
CALCITRIOL	4	(3)		(4)		
CARBON TETRACHLORIDE	9					
CHOLECALCIFEROL	6	(5)	(6)			
CISPLATIN	6	(7)	(8)			
CORBADRINE	1					
CYCLOSPORIN A	7	(9)		(10)		
DICLOFENAC	4					
FENOFIBRATE	5	(11)			(12)	
FLUOCINOLONE ACETONIDE	4	(13)		(14)		
IBUPROFEN	7	(15)			(16)	
INDOMETHACIN	9	(17)				
LEAD (II) ACETATE	7					
LIPOPOLYSACCHARIDE E. COLI O55-B5	14	(18)				
LY-294002	7	(19)				
MEBENDAZOLE	1	(20)				
MELOXICAM	4	(21)				
N,N-DIMETHYLFORMAMIDE	6					
N-NITROSODIETHYLAMINE	4					
NYSTATIN	4					
PRALIDOXIME CHLORIDE	4					
PROCARBAZINE	4	(22)	(23)			
PROCHLORPERAZINE	1				(24)	
ROFECOXIB	7	(25)				
SIROLIMUS	13	(26)	(27)			(28)
TRICHOSTATIN A	133	(29)				
VORINOSTAT	8	(30)		(31)		
**CASE 2**						
15-DELTA PROSTAGLANDIN J2	4	(32)				
ANISOMYCIN	1	(33)			(34)	
CEPHAELINE	1	(35)				
FLUPHENAZINE	4				(36)	
LY-294002	6	(19)				
MENADIONE	1	(37)		(38)		
MOMETASONE	1			(39)		
NICLOSAMIDE	1	(40)				
PARTHENOLIDE	1	(41)				
PERHEXILINE	4					
PHENOXYBENZAMINE	1					(42)
PROCHLORPERAZINE	4				(24)	
PUROMYCIN	1					
PYRVINIUM	4	(43)				
RIFABUTIN	1					
SIROLIMUS	5	(26)	(27)			(28)
SPIRONOLACTONE	5					
THIORIDAZINE	12	(44)			(45)	
TRICHOSTATIN A	67	(29)				
VALPROIC ACID	5	(46)			(47)	
VORINOSTAT	5	(30)		(31)		

[a]References indicate any kind of study relating the selected drugs and diseases.
[b]30 drugs with higher score and 10 more abundant.
[c]Times that the drug appears in the whole list of drug experiment entries.

1. Brunstein,M.G., Ghisolfi,E.S., Ramos,F.L.P. and Lara,D.R. (2005) A clinical trial of adjuvant allopurinol therapy for moderately refractory schizophrenia. *J. Clin. Psychiatry*, **66**, 213–219.

2. Yip,K.W., Mao,X., Au,P.Y.B., Hedley,D.W., Chow,S., Dalili,S., Mocanu,J.D., Bastianutto,C., Schimmer,A. and Liu,F.-F. (2006) Benzethonium chloride: a novel anticancer agent identified by using a cell-based small-molecule screen. *Clin. Cancer Res.*, **12**, 5557–69.

3. Segovia-Mendoza,M., Díaz,L., González-González,M.E., Martínez-Reza,I., García-Quiroz,J., Prado-Garcia,H., Ibarra-Sánchez,M.J., Esparza-López,J., Larrea,F. and García-Becerra,R. (2014) Calcitriol and its analogues enhance the antiproliferative activity of gefitinib in breast cancer cells. *J. Steroid Biochem. Mol. Biol.*

4. Kole,L., Cantrell,W. and Elewski,B. (2014) A randomized, double-blinded trial evaluating the efficacy and tolerability of vectical ointment (calcitriol 3 mcg/g ointment) when compared to betamethasone diproprionate ointment (64 mg/g) in patients with nail psoriasis. *J. Drugs Dermatol.*, **13**, 912–5.

5. Tokar,E.J. and Webber,M.M. (2005) Cholecalciferol (vitamin D3) inhibits growth and invasion by up-regulating nuclear receptors and 25-hydroxylase (CYP27A1) in human prostate cancer cells. *Clin. Exp. Metastasis*, **22**, 275–84.

6. Nakayama,J., Kiryu,H., Urabe,K., Matsuo,S., Shibata,S., Koga,T. and Furue,M. Vitamin D3 analogues improve café au lait spots in patients with von Recklinghausen's disease: experimental and clinical studies. *Eur. J. Dermatol.*, **9**, 202–6.

7. Florea,A.-M. and Büsselberg,D. (2011) Cisplatin as an anti-tumor drug: cellular mechanisms of activity, drug resistance and induced side effects. *Cancers (Basel).*, **3**, 1351–71.

8. Yazici,N., Varan,A., Akalan,N., Söylemezoğlu,F., Zorlu,F., Kutluk,T., Akyüz,C. and Büyükpamukçu,M. (2011) Diencephalic tumors in children: a 30-year experience of a single institution. *Childs. Nerv. Syst.*, **27**, 1251–6.

9. Kawahara,T., Kashiwagi,E., Ide,H., Li,Y., Zheng,Y., Ishiguro,H. and Miyamoto,H. (2015) The role of NFATc1 in prostate cancer progression: Cyclosporine A and tacrolimus inhibit cell proliferation, migration, and invasion. *Prostate*.

10. Johnson,J.L., West,D.A. and Haggstrom,A.N. (2015) Pyoderma gangrenosum associated with an aseptic splenic abscess in a patient with neurofibromatosis. *Pediatr. Dermatol.*, **32**, 113–7.

11. Han,D.-F., Zhang,J.-X., Wei,W.-J., Tao,T., Hu,Q., Wang,Y.-Y., Wang,X.-F., Liu,N. and You,Y.-P. (2015) Fenofibrate induces G0/G 1 phase arrest by modulating the PPARα/FoxO1/p27(kip) pathway in human glioblastoma cells. *Tumour Biol.*

12. Priestley,R.S., Nickolls,S.A., Alexander,S.P.H. and Kendall,D.A. (2014) A potential role for cannabinoid receptors in the therapeutic action of fenofibrate. *FASEB J.*, 10.1096/fj.14-263053.

13. Schwarz,J.A., Viaje,A. and Slaga,T.J. (1977) Fluocinolone acetonide: a potent inhibitor of mouse skin tumor promotion and epidermal DNA synthesis. *Chem. Biol. Interact.*, **17**, 331–47.

14. Hix,E., Gustafson,C.J., O'Neill,J.L., Huang,K., Sandoval,L.F., Harrison,J., Clark,A. and Feldman,S.R. (2013) Adherence to a five day treatment course of topical fluocinonide 0.1% cream in atopic dermatitis. *Dermatol. Online J.*, **19**, 20029.

15. Akrami,H., Aminzadeh,S. and Fallahi,H. (2014) Inhibitory effect of ibuprofen on tumor survival and angiogenesis in gastric cancer cell. *Tumour Biol.*

16. Chen,H., Jacobs,E., Schwarzschild,M.A., McCullough,M.L., Calle,E.E., Thun,M.J. and Ascherio,A. (2005) Nonsteroidal antiinflammatory drug use and the risk for Parkinson's disease. *Ann. Neurol.*, **58**, 963–7.

17. Chiou,S.-K., Hoa,N., Hodges,A., Ge,L. and Jadus,M.R. (2014) Indomethacin promotes apoptosis in gastric cancer cells through concomitant degradation of Survivin and Aurora B kinase proteins. *Apoptosis*, **19**, 1378–88.

18. Philipp,J., Dienst,A., Unruh,M., Wagener,A., Grunow,A., Engert,A., Fries,J.W.U. and Gottstein,C. (2003) Soluble tissue factor induces coagulation on tumor endothelial cells in vivo if coadministered with low-dose lipopolysaccharides. *Arterioscler. Thromb. Vasc. Biol.*, **23**, 905–10.

19. Venkatesh,H.S., Chaumeil,M.M., Ward,C.S., Haas-Kogan,D.A., James,C.D. and Ronen,S.M. (2012) Reduced phosphocholine and hyperpolarized lactate provide magnetic resonance biomarkers of PI3K/Akt/mTOR inhibition in glioblastoma. *Neuro. Oncol.*, **14**, 315–25.

20. Bai,R.-Y., Staedtke,V., Rudin,C.M., Bunz,F. and Riggins,G.J. (2014) Effective treatment of diverse medulloblastoma models with mebendazole and its impact on tumor angiogenesis. *Neuro. Oncol.*

21. Dong,X., Li,R., Xiu,P., Dong,X., Xu,Z., Zhai,B., Liu,F., Jiang,H., Sun,X., Li,J., *et al.* (2014) Meloxicam executes its antitumor effects against hepatocellular carcinoma in COX-2-dependent and -independent pathways. *PLoS One*, **9**, e92864.

22. Wick,W., Wiestler,B. and Platten,M. (2015) Treatment of anaplastic glioma. *Cancer Treat. Res.*, **163**, 89–101.

23. Ater,J.L., Zhou,T., Holmes,E., Mazewski,C.M., Booth,T.N., Freyer,D.R., Lazarus,K.H., Packer,R.J., Prados,M., Sposto,R., *et al.* (2012) Randomized study of two chemotherapy regimens for treatment of low-grade glioma in young children: a report from the Children's Oncology Group. *J. Clin. Oncol.*, **30**, 2641–7.

24. Lacasse,H., Perreault,M.M. and Williamson,D.R. (2006) Systematic review of antipsychotics for the treatment of hospital-associated delirium in medically or surgically ill patients. *Ann. Pharmacother.*, **40**, 1966–73.

25. McKenzie,B.A., Zemp,F.J., Pisklakova,A., Narendran,A., McFadden,G., Lun,X., Kenchappa,R.S., Kurz,E.U. and Forsyth,P.A. (2015) In vitro screen of a small molecule

inhibitor drug library identifies multiple compounds that synergize with oncolytic myxoma virus against human brain tumor-initiating cells. *Neuro. Oncol.*

26. Chheda,M.G., Wen,P.Y., Hochberg,F.H., Chi,A.S., Drappatz,J., Eichler,A.F., Yang,D., Beroukhim,R., Norden,A.D., Gerstner,E.R., *et al.* (2014) Vandetanib plus sirolimus in adults with recurrent glioblastoma: results of a phase I and dose expansion cohort study. *J. Neurooncol.*

27. Weiss,B., Widemann,B.C., Wolters,P., Dombi,E., Vinks,A., Cantor,A., Perentesis,J., Schorry,E., Ullrich,N., Gutmann,D.H., *et al.* (2014) Sirolimus for progressive neurofibromatosis type 1-associated plexiform neurofibromas: a Neurofibromatosis Clinical Trials Consortium phase II study. *Neuro. Oncol.*

28. Hunt,K.M., Herrmann,J.L., Andea,A.A., Groysman,V. and Beckum,K. (2014) Sirolimus-associated regression of benign lymphangioendothelioma. *J. Am. Acad. Dermatol.*, **71**, e221–2.

29. Höring,E., Podlech,O., Silkenstedt,B., Rota,I.A., Adamopoulou,E. and Naumann,U. (2013) The histone deacetylase inhibitor trichostatin a promotes apoptosis and antitumor immunity in glioblastoma cells. *Anticancer Res.*, **33**, 1351–60.

30. Friday,B.B., Anderson,S.K., Buckner,J., Yu,C., Giannini,C., Geoffroy,F., Schwerkoske,J., Mazurczak,M., Gross,H., Pajon,E., *et al.* (2012) Phase II trial of vorinostat in combination with bortezomib in recurrent glioblastoma: a north central cancer treatment group study. *Neuro. Oncol.*, **14**, 215–21.

31. Kavanaugh,S.M., Kavanaugh,S.A., White,L.A. and Kolesar,J.M. (2010) Vorinostat: A novel therapy for the treatment of cutaneous T-cell lymphoma. *Am. J. Health. Syst. Pharm.*, **67**, 793–7.

32. Khan,S.A., Virtanen,S., Kallioniemi,O.P., Wennerberg,K., Poso,A. and Kaski,S. (2014) Identification of structural features in chemicals associated with cancer drug response: a systematic data-driven analysis. *Bioinformatics*, **30**, i497–504.

33. You,P., Xing,F., Huo,J., Wang,B., Di,J., Zeng,S. and Liu,J. (2013) In vitro and in vivo evaluation of anisomycin against Ehrlich ascites carcinoma. *Oncol. Rep.*, **29**, 2227–36.

34. Pena,R.R., Pereira-Caixeta,A.R., Moraes,M.F.D. and Pereira,G.S. (2014) Anisomycin administered in the olfactory bulb and dorsal hippocampus impaired social recognition memory consolidation in different time-points. *Brain Res. Bull.*, **109**, 151–7.

35. Muhammad,I., Dunbar,D.C., Khan,S.I., Tekwani,B.L., Bedir,E., Takamatsu,S., Ferreira,D. and Walker,L.A. (2003) Antiparasitic alkaloids from Psychotria klugii. *J. Nat. Prod.*, **66**, 962–7.

36. Tardy,M., Huhn,M., Engel,R.R. and Leucht,S. (2014) Fluphenazine versus low-potency first-generation antipsychotic drugs for schizophrenia. *Cochrane database Syst. Rev.*, **8**, CD009230.

37. Suresh,S., Raghu,D. and Karunagaran,D. (2013) Menadione (Vitamin K3) induces apoptosis of human oral cancer cells and reduces their metastatic potential by modulating the

expression of epithelial to mesenchymal transition markers and inhibiting migration. *Asian Pac. J. Cancer Prev.*, **14**, 5461–5.

38. Perez-Soler,R., Zou,Y., Li,T. and Ling,Y.H. (2011) The phosphatase inhibitor menadione (vitamin K3) protects cells from EGFR inhibition by erlotinib and cetuximab. *Clin. Cancer Res.*, **17**, 6766–77.

39. Roy,C. and Chakrabarty,J. (2013) Development and Validation of a Stability-Indicating RP-HPLC Method for the Simultaneous Determination of Phenoxyethanol, Methylparaben, Propylparaben, Mometasone Furoate, and Tazarotene in Topical Pharmaceutical Dosage Formulation. *Sci. Pharm.*, **81**, 951–67.

40. Li,Y., Li,P.-K., Roberts,M.J., Arend,R.C., Samant,R.S. and Buchsbaum,D.J. (2014) Multi-targeted therapy of cancer by niclosamide: A new application for an old drug. *Cancer Lett.*, **349**, 8–14.

41. Al-Fatlawi,A.A., Al-Fatlawi,A.A., Irshad,M., Rahisuddin and Ahmad,A. (2015) Effect of parthenolide on growth and apoptosis regulatory genes of human cancer cell lines. *Pharm. Biol.*, **53**, 104–9.

42. Caine,M., Perlberg,S. and Meretyk,S. (2002) A placebo-controlled double-blind study of the effect of phenoxybenzamine in benign prostatic obstruction. 1978. *J. Urol.*, **167**, 1101.

43. Xu,W., Lacerda,L., Debeb,B.G., Atkinson,R.L., Solley,T.N., Li,L., Orton,D., McMurray,J.S., Hang,B.I., Lee,E., *et al.* (2013) The antihelmintic drug pyrvinium pamoate targets aggressive breast cancer. *PLoS One*, **8**, e71508.

44. Kang,S., Dong,S.M., Kim,B.-R., Park,M.S., Trink,B., Byun,H.-J. and Rho,S.B. (2012) Thioridazine induces apoptosis by targeting the PI3K/Akt/mTOR pathway in cervical and endometrial cancer cells. *Apoptosis*, **17**, 989–97.

45. Ichikawa,J., Dai,J., O'Laughlin,I.A., Fowler,W.L. and Meltzer,H.Y. (2002) Atypical, but not typical, antipsychotic drugs increase cortical acetylcholine release without an effect in the nucleus accumbens or striatum. *Neuropsychopharmacology*, **26**, 325–39.

46. Yang,W., Zhao,X., Pei,F., Ji,M., Ma,W., Wang,Y. and Jiang,G. (2015) Activation of the intrinsic apoptosis pathway contributes to the induction of apoptosis in hepatocellular carcinoma cells by valproic acid. *Oncol. Lett.*, **9**, 881–886.

47. Luo,Z., Fang,Y. and Zhang,L. (2015) The effects of antiepileptic drug valproic acid on apoptosis of hippocampal neurons in epileptic rats. *Pak. J. Pharm. Sci.*, **28**, 319–24.
